# Does Poland’s agri-food industry gain comparative advantage in trade with non-EU countries? Evidence from the transatlantic market

**DOI:** 10.1371/journal.pone.0274692

**Published:** 2022-09-16

**Authors:** Karolina Pawlak, Luboš Smutka

**Affiliations:** 1 Faculty of Economics, Department of Economics and Economic Policy in Agribusiness, Poznan University of Life Sciences, Poznan, Poland; 2 Faculty of Economics and Management, Department of Trade and Finance, Czech University of Life Sciences Prague, Prague, Czech Republic; Jain University, INDIA

## Abstract

Accession of Poland to the European Single Market generated trade creation and diversion effects, which in turn resulted in a high degree of concentration of the Polish foreign trade in agri-food products with other EU countries. On the one hand, a high share of export to the markets of countries with a stable market economy is a confirmation of the Polish agri-food sector’s capacity to compete on the foreign markets. On the other hand, when considering limited capability to increase food demand in the EU it provides grounds for the assumption that further export expansion to a considerable extent will depend on the potential expansion of sale to non-EU markets. In this context significant issues include diversification of target markets and search for prospective markets outside the EU, while they also determine directions of an advantageous export specialization. In the period up to 2021 the USA was the fourth non-EU export partner of Poland in the agri-food sector after the United Kingdom, Ukraine and Russia. The aim of this paper is to evaluate the level of comparative advantages of the Polish agri-food sector on the EU and US markets in 2004–2021 using Widodo’s products mapping technique. The study used statistical data from the ComExt database of the European Statistical Office (Eurostat). The conducted studies showed Polish export specializations in the transatlantic trade, as confirmed by high comparative advantages, as well as a positive and steadily improving trade balances for food preparations, including meat, cereal, fruit and vegetable, as well as confectionery preparations, and less processed animal origin products (meat and offal as well as dairy produce). Poland had a disadvantageous competitive position in trade of products complementary in relation to domestic production, which import was necessary. In view of the comparative cost theory the directions of the realized export specialization were rational and should be maintained. While in trade with the other EU countries the competitive position of the Polish agri-food sector was weakened, it was strengthened on the US market. In view of the considerable EU market saturation the improved competitive capacity in the non-EU markets is a positive development, which is a promising finding particularly in the long-term perspective.

## Introduction

Agriculture and the food industry are strategic branches of national economies in countries regardless of their development levels. It is determined not only by the character of produced commodities and goods and their role in the assurance of food security, but also their share in the generation of the gross domestic product, development of the labor market as well as their importance in international trade [1–5]. In 2020 the value of the world export of agri-food products was 1.6 trillion US dollars, which accounted for almost 10% total export of goods. Almost 15% trade in agri-food products resulted from export of agricultural raw materials, while the rest of export was connected with products of the food industry [6]. The ongoing intensification of trade and a growing importance of import in meeting the demand for food, especially in food-deficit countries, have been observed since the mid-1980s. According to D’Odorico et al. [7], around 23% of world food production is traded, while as it results from estimates by Fader et al. [8] 16% of the global population consumes food that is available on the domestic markets thanks to international trade. The importance of a country’s trade openness to an improvement of food availability and achieving food security was proved e.g. by Dithmer nad Abdulai [9], Tinta et al. [10], Ly et al. [11] and Sun and Zhang [12].

The agri-food trade traditionally occupies an important position in the structure of Poland’s foreign trade, while the effect of trade creation generated by the accession to the European Single Market caused a significant increase in the importance of agricultural trade in the total volume of Poland’s foreign trade. In the years 2004–2021 the share of agri-food export in the total export of goods increased by almost 4.5 percentage points and reached 13.1% [13]. Following Poland’s accession to the EU, export became a significant factor determining the level of equilibrium on the agricultural markets and influencing the situation in many branches of the food industry, particularly fish, tobacco, coffee and tea processing as well as confectionery industries, which are characterized by strong export orientation, selling over 50% of their production on foreign markets. Moreover, in the tobacco and confectionery industries, as well as meat, dairy and vegetable industries export helped to break down the internal demand barrier. In turn, import not only facilitated better utilization of the production potential and improved competitiveness of many branches (e.g. processing of fish, vegetable and fruit, coffee, cocoa, chocolate and spices), but also contributed to increased consumption and improved the quality of nutrition [14].

A characteristic feature of the Polish foreign trade in agri-food products is connected with a high degree of concentration of trade with the other EU countries. As a consequence of the trade diversion effect after Poland’s incorporation into the EU customs union, the share of intra-regional trade in the Polish agri-food export increased to over 70%, while for import it reached almost 80% (excluding the United Kingdom, which left the EU in 2021) [13]. On the one hand, such a high share of export to more economically developed countries with stable market economies indicates the competitive capacity of the Polish agri-food sector on foreign markets. However, in view of the limited potential to increase the EU food demand it may be expected that a further development of this sector of the economy will depend, among other things, on the potential to expand trade to non-EU markets. In turn, diversification of markets and effective placement of products both on the EU markets and on non-EU markets will force Polish producers and exporters to face competitive pressure from other food suppliers.

In the period up to 2021 the USA ranked fourth, after the United Kingdom, Ukraine and Russia, as Poland’s non-EU export partner in the agri-food sector [13]. At the same time Poland was the greatest US trade partner among the Central and Eastern European countries. The EU countries and the USA are important mutual economic partners, while at the same time being competitors on the world market. Bilateral relations of these countries affect their international position. Although negotiations on the establishment of the Transatlantic Trade and Investment Partnership (TTIP) were unsuccessful, opportunities for enhanced mutual economic relations and strengthening of the international competitive position of these economies were considered to be associated with establishment of close institutional cooperation and the EU-USA free trade area. It needs to be remembered that these economies face growing pressure from the emerging economies, including BRICS, gaining in geopolitical importance and economic strength [15–19]. It results from the studies by Francois et al. [20] and Bureau et al. [21] that the establishment of the free trade area between the EU and the US would boost their mutual trade, giving both economies an opportunity to increase their market shares and strengthen their international competitive position. As it was shown by Hagemejer et al. [22], the trade creation effect may also be applied to the Polish-American agri-food trade.

In this context a question may be posed whether the American market might become a prospective market for the Polish agri-food sector, facilitating diversification of export markets outside the EU. It also needs to be remembered that the development of effective sales in the US market would force Polish producers not only to face competition from American farmers, characterized by more favorable ratios between production factors and a greater scale of land concentration, resulting in a higher productivity level. It would also require them to compete with producers from the other EU countries with a greater competitive capacity of the agricultural sector, such as Germany, the Netherlands, France, Denmark or Belgium [23], as well as from non-EU countries benefiting from lower labor costs or better natural conditions for agricultural production. In view of the above, a key issue would be to identify highly competitive groups of products, which may become the basis for an advantageous export specialization. Thus, the aim of this paper is to evaluate the level of comparative advantages of the Polish agri-food sector on the EU and US markets in 2004–2021.

The paper is motivated by the need to define prospective non-EU markets and identify advantageous export specializations, which may give Poland the foundations for maximization of gains from agri-food trade when seeing the limited capacity to improve the competitiveness on the European Single Market. It should be stressed here that empirical studies on trade competitiveness of the Polish agri-food sector are quite numerous and extensive. However, most of them assess the level and duration of Poland’s comparative trade advantages in intra-EU trade [24–29] or on the global market [30–32]. Based on those investigations it can be concluded that agri-food trade of Poland increased with its accession to the EU, while its comparative advantages in relation to both the European Single Market and the global market had been constantly improving in the first years of Poland’s membership in the EU. Higher and more stable relative trade advantages were found for labor-intensive agricultural raw materials or food items produced at lower cost resulting mainly from lower raw material prices and lower processing margins. The competitive position of the Polish agri-food sector was also investigated in trade with some non-EU countries, including Asian or South American markets [33,34], but–excluding the study by Pawlak [35]–there is a lack of analyses referring to the US market, which potentially can be a prospective location for Polish exports. This needs to be considered especially when the other key non-EU trading partners for Poland include those, with which there is currently a relatively high non-market risk in trade. It is uncertain how the regulatory cooperation between the EU and the UK will work after Brexit, while Russia and Ukraine are currently at war. An additional motivation for our research is that in a long run, when the trade creation effect was losing its strength, Poland’s competitive position on the European Single Market has weakened, while its strengthening was noted in trade with third countries [36]. This shows that rethinking the geographical and assortment structure of Poland’s agri-food trade is of key importance in order to maintain the country’s strong competitive position.

The novelty and contributions of this paper are fourfold. Firstly, up to our knowledge this is the first study offering a comparative analysis of the international competitive position of the Polish agri-food sector on the EU and US markets, which are the biggest players in the world trade in agri-food products. Secondly, we applied the products mapping technique proposed by Widodo [37], which is a more comprehensive method to evaluate competitiveness than the Balassa approach [26,27,30,38–40] or the Vollrath approach [24,25,29,31,41,42] used by most existing studies. Thirdly, based on the identification of Poland’s export specializations and verifying whether the US market may be a prospective one, a more utilitarian value of the study appears to consist in giving recommendations for decision makers on possible changes in the export structure to optimize gains from trade. Finally, we analyzed the trade pattern and comparative advantages of the agri-food sector in Poland, being the greatest trade player among the Central and Eastern European countries, which experienced a significant transformation of trade under the country’s EU membership. Poland’s case study offers a possibility to observe a trade adjustment path and long-term changes in the competitive capacity in a country improving its trade openness under accession to the custom union, both taking advantage from the trade creation effect and facing the problem of deep trade concentration within that common market. From that point of view the results of our study can be of broader relevance for the audience interested in sector-specific analyses of trade developments under a changing institutional environment. In this way, the research results contribute to the existing literature in the field of international trade, international competitiveness and economic integration at the mesoeconomic level.

The rest of the paper is organized as follows. A literature review focuses on the existing approaches to the comparative advantage measurement and presents some achievements in the field focusing on agri-food trade and Poland’s case. Next, we explain the data and methodology used. Then, we discuss research results starting with Poland’s agri-food trade pattern, which is the background for the discussion on the distribution of comparative advantages of the Polish agri-food sector on the EU and US markets. The last section sums up and concludes the study.

### Literature review

The concept of international competitiveness is ambiguous, as with reference to various economic theories it is defined in numerous ways for both short- and long run perspectives [43–46]. One of the most commonly known points of view states that a competitive agri-food industry is able to achieve profitable gain and maintain the market share in export markets, in which the industry is active [43,47]. In order to measure the competitive performance, trade-related indicators such as market performance, trade balance and the revealed comparative advantage indicators are often used. The classical theory of trade suggests that a country’s competitiveness is based on the concept of comparative advantage, which is one of the oldest and most important concepts in economics [44]. It should be emphasized here that even the advances in modern trade theories did not diminish the importance of the Ricardian trade model, indicating relative differences in production costs to be the reason for beneficial export specialization when explaining the patterns of agricultural trade [35]. Therefore, the concept of comparative advantage has often been employed in the research of international competitiveness of agri-food sectors. Among others, this approach was used by Bojnec [41], Bavorova [48], Fertő and Hubbard [42], Pawlak and Poczta [24], Wijnands et al. [47,49], Pawlak et al. [25], Kita and Poczta [50], Ishchukova and Smutka [51], Rytko [26], Carraresi and Banterle [38], Juchniewicz and Lukiewska [39], Bojnec and Fertő [30,40], Cimpoies [52], Kita [33,34], Wijnands and Verhoog [53], Smutka et al. [54], Szczepaniak [27,31], Ortikov et al. [55], Verter et al. [56], Zdrahal et al. [57] and Pawlak [19,35,58,59]. All those studies refer to the competitive position of the agri-food sector of individual countries or groups of countries either on the world market or in specific bilateral relations.

Two popular trade related indices employed in the competitiveness research are export market shares and the Balassa Revealed Comparative Advantage Index (RCA), which is a ratio of the share of export of the analyzed product in world/regional export to the share of export of the entire sector in world/regional export [60]. These indicators were used e.g. by Carraresi and Banterle [38] to evaluate the competitive performance of the food industry in individual EU countries on the intra-EU market in 1995–2011 and to assess the effects of the EU enlargement and the 2008–09 economic crisis on competitiveness of individual countries. They found divergent trends in competitive performance of the food industry in countries under investigation. German and Dutch food industries have profited the most from the EU enlargement. A significant competitive performance was noted in the case of food industries in Central and Eastern European countries, while food industries in France and Belgium have lost their competitiveness. At the same time the competitive position of Italy and Spain showed a substantial competitive stasis. Competitiveness of Polish and Slovak agri-food products in intra-EU trade was also determined by Rytko [26]. Calculations of export shares and the Balassa indices let her conclude that contrary to Slovakia, Poland has had comparative advantages on the European Single Market since 2005. Those two indicators along with the Trade Coverage Ratio (TC), i.e. the export-import ratio in a given sector, were employed by Juchniewicz and Lukiewska [39] to present the competitive position of the food industry of the EU on the global market in 2000–2013. According to that study, the EU as a whole did not have comparative advantages in world food trade. Such countries as Argentina, Brazil, Australia, Thailand, Indonesia, India, Canada and the US appeared to be much more competitive food exporters, which is in line with the results by Wijnands et al. [49] and Wijnands and Verhoog [53].

The RCA indices were also used by Szczepaniak [27] to assess comparative advantages in Poland’s food export to the EU in 2003–2015. It was shown that a significant improvement in the competitive position of Polish food producers on the EU market was observed, while the strongest revealed comparative advantages were held in trade in animal products and prepared foodstuffs. In turn, Bojnec and Fertő [40] employed the RCA indices to discuss changes in agri-food export competitiveness of the EU countries in the global market in 2000–2011. They demonstrated that the EU enlargements towards the East have contributed to a slight increase in comparative advantages gained by the EU agri-food industry as a whole in the world market. In terms of individual countries the most competitive EU member states were the Netherlands, France and Spain. As in the study by Carraresi and Banterle [38] referring to the intra-EU trade, Bojnec and Fertő [40] noted that the new EU member states were successful in the agri-food export to the global market. Based on the Balassa index, Bojnec and Fertő [30] investigated also the duration of comparative advantage in the EU agri-food exports on the global market. They indicated that in 2000–2011 most of the old EU member states experienced a greater number of agri-food products with a longer duration of comparative advantages than most of the new EU member states. Using an analogous empirical method, Bojnec and Fertő [28] analyzed also the dynamics in revealed comparative advantage of the new EU member states in intra-EU trade in agri-food products. They found that in the countries under investigation the levels of both trade and revealed comparative advantages in agri-food products increased after joining the EU. However, some catching-up difficulties were observed in value added products.

Since a weakness of the Balassa index stems from the estimation of comparative advantages only based on the value of export, to ensure objectivity of the results of conducted analyses many researchers of international competitiveness use Vollrath’s modified version of the original Balassa’s index, which is called the Revealed Competitiveness Index (RC). The RC index is a difference of logarithmic forms of the Balassa index (RCA) and the index of revealed comparative advantages in imports (RMA), being a ratio of the share of import of the analyzed product in world/regional import to the share of import of the entire sector in world/regional import [61]. Using the approach proposed by Vollrath and referring both to the situation in export and import, Fertő and Hubbard [42] analyzed the comparative advantage of the Hungarian agri-food sector in relation to the EU market in 1992–1998. They found that Hungary had a comparative advantage in export of 11 out of 22 product groups under investigation, including cereals, meat, sugar and live animals; however, the competitive position of the country weakened over the investigated period. A similar method was applied by Szczepaniak [31] to assess the competitiveness of Polish agri-food sector in world trade in 2004–2017. She combined comparative advantage analysis with the Lafay Trade Balance Index (TBI) to show that during the analyzed period Poland improved its competitive position on the world market. According to that study, in 2017 agri-food products with the strongest comparative advantages and a positive trade balance accounted for 55.5% of total Polish export, which was by 12.8 percentage points more than in 2004. Both Hungarian and Polish agri-food sectors along with the agri-food sectors in other Central European and Balkan countries were also investigated by Bojnec and Fertő [29] in terms of the duration of relative trade advantages on the EU-15 markets. Contrary to their previous study [28], they employed the Vollrath approach and found that the EU enlargement increased competitive pressures, while higher and more stable trade advantages were reached by the analyzed countries for agricultural raw materials rather than differentiated products. Moreover, they found that Poland was among the countries with the highest duration of agri-food trade advantages in differentiated products, which shows its potential in trade with the EU-15 countries.

An alternative way to consider both export and import flows to evaluate international competitiveness of the agri-food sector is to compare the absolute values of RCA and RMA indices rather than natural logarithms. The simple difference between the RCA and RMA indices is known as the Relative Trade Advantage Index (RTA) and all these three indices have been extensively used in analyses of competitiveness. Cimpoies [52] employed such an approach to assess competitiveness of Moldova’s agri-food sector on the EU market in 2001–2014. Meat and edible meat offal, meat preparations, vegetables, beverages, spirits and vinegar, tobacco and manufactured tobacco substitutes were among commodities showing the strongest competitive position. In turn, Wijnands et al. [47] assessed the competiveness performance of the Swiss food industry against selected EU countries based on trade related indicators such as world market shares and the RTA index, which were accompanied by economic indicators, including real turnover growth rate and labor productivity growth rate. They concluded that the Swiss food and beverage industry appears to be highly competitive compared to the benchmark EU countries. A corresponding method was also used by Wijnands et al. [49] and Wijnands and Verhoog [53] to evaluate competitiveness of the EU food industry in relation to the USA, Australia, Brazil and Canada. It results from those analyses that the EU competitive position weakened in 2003–2012, while the Brazilian food sector remained the most competitive and the US sector grew stronger. Following the approach given by Wijnands et al. [49] when considering the period of 2007–2016, a deterioration of competitive performance of the EU food industry on the world market against the USA and Canada as benchmark countries was also reported by Pawlak [59].

To avoid double counting which would lead to biased RCA and RMA values, it is advisable to exclude from the values of world/regional trade (serving as the benchmark) the subject country’s export [62]. Taking into account this adjustment Bojnec [41] used the RCA, RMA and RTA indicators to show diversification of revealed comparative advantages of the agri-food sectors in selected countries and regions in the world trade. Those adapted indicators of comparative advantages were also employed by Bavorova [48], who revealed the comparative disadvantage of Czech sugar producers on the world market. The same method was applied by Pawlak and Poczta [24] and Pawlak et al. [25] to evaluate the competitive position of the agri-food sectors of Poland and the other new EU member states on the European Single Market. It results from those analyses that after accession to the EU Poland gained comparative advantages in key groups of agri-food products exported to the other EU countries (dairy products, meat and edible meat offal, meat preparations, preparations of fruit and vegetables, sugars and sugar confectionery), reaching the strongest competitive position in labor-intensive products, which taking into consideration the cost factor it is in line with the Heckscher-Ohlin-Samuelson theorem. Hungary was another country enjoying a relatively strong competitive position in intra-EU trade. In both countries thanks to lower processing margins compared to those in Western Europe, a relatively high level of comparative advantages on the European Single Market were observed for products characterized by higher processing rates.

The competitive position of the Polish agri-food sector was also investigated in relation to the non-EU markets. Kita and Poczta [50], using a similar set of indicators as the previously mentioned authors, performed an analysis of Poland’s comparative advantages on the Commonwealth of Independent States (CIS) market. They stated that in 2000–2009 animal origin products exported from Poland to the CIS countries had a stronger competitive position than those of plant origin. The highest comparative advantages were found in trade in live animals as well as meat and meat preparations with Russia and Ukraine. Using that methodology, a favorable competitive position of Polish exporters of animal origin products was also shown in relation to selected Asian markets, including China, Japan and the Republic of Korea [34]. On the other hand, due to numerous non-tariff barriers to trade and a high level of food self-sufficiency of the trading partners, Poland was less successful when competing on the MERCOSUR agri-food market [33].

The RCA and related indicators are simple measures of international competitiveness of agri-food sectors which allow us to compare the subject countries. Another advantage of using trade based indicators is that the costs of marketing and transportation are also included [62]. However, a weakness of these methods is related to the non-symmetric distribution [27]. Since RCA results in an output which cannot be compared on both sides of unity (its neutral value), several modifications of the Balassa index have been developed to eliminate this problem. One of the measures suggested is the so-called Revealed Symmetric Comparative Advantage (RSCA) [63]. The normalized RCA index, as (RCA-1)/(RCA+1), falls between -1.0 and +1.0 and as it was tested by Laursen [64] it proved to be the best measure of comparative advantage. Although De Benedictis and Tamberi [65] pointed out that such a transformation of the RCA index does not yield major interpretation benefits, it is used together with the Lafay trade balance index (TBI) to build two-dimension matrices facilitating analyses of countries’ comparative advantages and identify key exported products that contribute the most to the trade balance and are a source of gains from trade. That “products mapping” method originally designed by Widodo [37] has been employed in a series of studies.

Using the Widodo “products mapping” tool Ishchukova and Smutka [51] studied export specialization and competitive performance of the Russian agri-food sector in trade with Africa, the Americas, Asia, CIS and the EU countries in 1998–2010. They found that in the case of most products Russia had comparative advantages on the CIS, EU and Asian markets, while in trade with Africa and the Americas comparative disadvantages were observed. It was also identified that products with the highest comparative advantages and positive trade balance amounted to around 50% of the total agri-food export value. At the same time products characterized by an unfavorable competitive situation (comparative disadvantage and negative trade balance) accounted for about 30% of export value, but more than 95% of total agri-food imports. The Widodo matrix was also applied by Smutka et al. [54] to specify changes in competitiveness of the Czech foreign trade in agri-food products in 2001–2015. It was demonstrated that Czech agri-food products were not competitive on the world market, but experienced comparative advantages at bilateral levels, especially in relation to the EU market. By constructing the Widodo matrices Ortikov et al. [55] observed that in 2000–2018 agri-food products exported from Uzbekistan were competitive with regard to Asian and CIS markets, while the ability to successfully compete on the other markets was limited. The same approach was also employed by Verter et al. [56] and Zdrahal et al. [57]. The former authors investigated the dynamics of comparative advantages in Nigerian agri-food trade with the EU in 1995–2017. Due to the decreasing number of agri-food products enjoying a strong competitive position accompanied by a positive trade balance both on the EU market and in world trade, it was recommended for Nigeria to increase investment and improve agricultural and trade policies in order to boost trade and at the same time increase its food self-sufficiency. Also Zdrahal et al. [57] focused on developing countries and analyzed trade performance and competitiveness of South Africa’s agri-food sector on the EU and African markets in 2005–2017. Their results supported the conclusion that in contrast to trade relations with the EU, South Africa gained comparative advantages in relation to the African market. However, a cause for concern may stem from the fact that no clear diversification was observed towards a larger number or new export specialization products that can contribute to the positive trade balance. Commodities playing such a role included fish, fruit and nuts.

The method proposed by Widodo [37] was also applied in the analyses of competitiveness of transatlantic trading partners. Pawlak [58] examined the pattern of comparative advantages of the EU and US agri-food sector on the world market in 1995–2015. It was found that the EU countries held comparative advantages in the export of animal origin products, while the USA had a strong competitive position in the export of cereals and preparations thereof, oil seeds and meat products. In the case of both economies these were the products representing the highest shares in total agri-food exports and generating a high and constantly increasing positive trade balance. Other studies referred to bilateral relations of the EU countries and the US. For instance, Pawlak [19] assessed the competitive position of the EU agri-food sector on the US market in 2010–2020. It results from that research that the comparative advantages gained by the EU in trade with the USA were sources of an advantageous export specialization. A corresponding conclusion was drawn with regard to Poland’s agri-food export to the US market [35]. It was stated that Poland attained high comparative advantages in products being export specializations and contributing to the positive trade balance, including products of the milling industry, meat preparations, cocoa and cocoa products, cereal preparations, sugar confectionery and dairy products. In turn, imports predominantly included products complementary to the domestic production with no comparative advantages. In line with the Ricardian principle of comparative advantage the commodity structure of Poland’s agri-food trade with the US was considered coherent.

As one indicator is not sufficient to assess the broad issue of competitiveness [45], the Widodo approach employing two indicators related to both the export and import situation of the subject country can be taken as a relatively simple, but at the same time comprehensive method to evaluate competitiveness. Even though a few studies have assessed the competitive position of Poland’s agri-food sector, mainly Vollrath’s approach and related indices were used, while simple descriptive analyses dominated at the same time. The product mapping technique in a synthetic and transparent form lets us consider both comparative advantages and the nature of trade balance, the use of which can raise some doubts [66] at the macroeconomic level, but is widely accepted at the mesoeconomic level [67]. Secondly, it results from the above literature review that most research evaluating competitiveness of the Polish agri-food sector referred to the European Single Market. Very few studies investigated relations with individual non-EU trading partners, whereas available studies covered the period up to 2010. Additionally, despite the relatively high importance of the US among Poland’s non-EU export partners–to our best knowledge–only one analysis of competitiveness performance of the Polish agri-food sector on the US market has been conducted to date [35]. There were also no studies comparing the international competitive position of the Polish agri-food sector on both transatlantic markets (the EU and US markets). Up to date there were only some comparative studies concerning the production potential and productivity of agriculture in Poland, the other EU countries and the USA, or similarity of the Polish agri-food export in relation to France and Germany on the EU, US and China markets, including those by Pawlak and Poczta [68], Pawlak et al. [23], Bajan et al. [69]. This is surprising, since such investigations may be valuable in the context of export structure diversification and the search for key alternative markets outside the EU, as well as the potential for reviving the transatlantic relations, that have stagnated after the suspension of the TTIP negotiations in 2016. Our paper fills this research gap and offers a dynamic assessment of comparative advantages of the Polish agri-food sector on the EU and US markets in 2004–2021 using the products mapping technique. Moreover, the utilitarian value of the study may be related to identifying Poland’s export specializations and thus formulating recommendations on the possible reorientation of the export structure to provide the highest possible gains from trade with both partners, as expected in the open economy model. The research may give grounds and justification for decision makers to take actions towards improving the policies enhancing international competitiveness of the Polish agri-food sector.

## Materials and methods

This study used statistical data from the ComExt database of the European Statistical Office (Eurostat). The timeframe of the research covers the years 2004 and 2021, which is sufficient to identify long-term changes in the pattern of comparative advantages, indicate export specializations and conclude on possible adjustments in the export structure to gain from trade with partners under investigation. Bilateral trade values were established based on mirror statistics, which means that the export value reported by country A to country B is equal in value to the import value reported by country B for the same trade flow. A similar methodological approach was previously employed e.g. by Guo [70], Hamanaka [71], Day [72], Javorsek [73], Markowicz and Baran [74], Naumov et al. [75] and Yurik et al. [76]. Trade values were expressed in euro at current prices. Comparative advantages were analysed at the 2-digit level of the Harmonized System (HS) nomenclature. This is a standard approach commonly applied in the literature on subject [19,25,31,38,52,55]. However, to be slightly more specific when looking for export specializations within those 2-digit groups based on the export shares, the 4-digit HS items with the highest export potential were identified.

The analysis of comparative advantages of the Polish agri-food sector was conducted applying the Balassa Revealed Comparative Advantage Index (RCA), the Vollrath Revealed Competitiveness Index (RC), the Revealed Symmetric Comparative Advantage Index (RSCA) and the Lafay Trade Balance Index (TBI). All those indicators were adapted to meet the requirements of the analysis of bilateral trade relations.

The Balassa Revealed Comparative Advantage Index (RCA) was determined from the formula [60]:

$$RCA_{ij}=RXA_{ij}=(X_{ij}/X_{ik})/(X_{nj}/X_{nk})$$ (1)

where: X–export, i–country under investigation, j–product (product group) under investigation, k–all goods, n–reference country (countries). Values of RCA above 1 indicate an advantageous competitive position, while lower ones demonstrate comparative disadvantage [60].

In order to make the competitiveness analysis more comprehensive and consider import flows when reasoning, the Vollrath Revealed Competitiveness Index (RC) was calculated as the difference between natural logarithms of the revealed comparative advantages in export (RCA) and import (RMA) [61]:

$$RC_{ij}=ln\;(RXA_{ij})–ln\;(RMA_{ij})$$ (2)

where:

$$RMA_{ij}=(M_{ij}/M_{ik})/(M_{nj}/M_{nk})$$ (3)

Positive RC values denote the existence of a comparative advantage, while negative values show its lack.

The Revealed Symmetric Comparative Advantages (RSCA) with values varying from -1.0 to +1.0, where negative and positive values reveal, respectively, an advantageous and disadvantageous competitive position, was determined as follows [63]:

$$RSCA_{ij}=(RCA_{ij}-1)/(RCA_{ij}+1)$$ (4)

The Lafay Trade Balance Index (TBI) was calculated according to the formula [77]:

$$TBI_{ij}=(X_{ij}-M_{ij})/(X_{ij}+M_{ij})$$ (5)

Similarly to RSCA, values of TBI fall within the range between -1.0 and +1.0. Positive values denote export specialization of a given country which is in the position of net exporter of a specific product (product group). In turn, negative values indicate a lack of specialization and the net importer position of an analyzed country.

**Fig 1:**
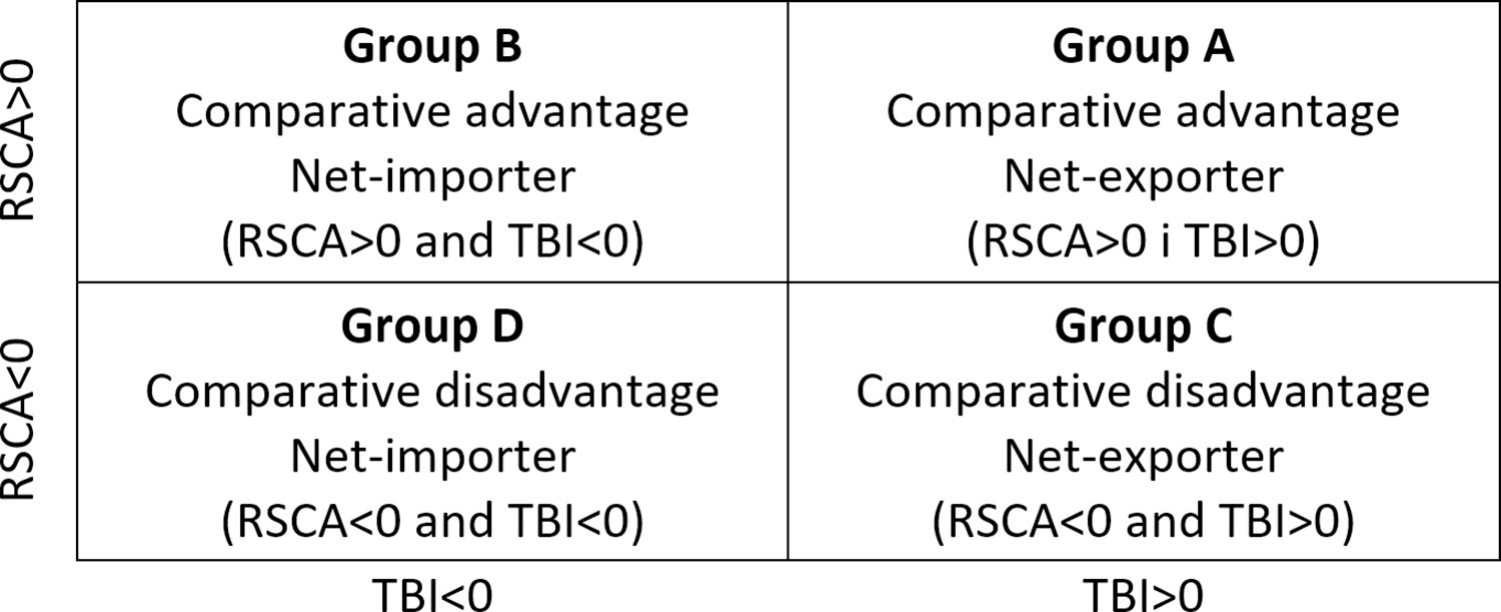
Products mapping by the RSCA and TBI values in line with the Widodo approach. Source: Widodo [37].

Following the Widodo approach [37] and based on the RSCA and TBI values, it was possible to develop matrices facilitating the division of the analysed set of products into four groups differing in the level of comparative advantages (RSCA) and the degree of export specialization (TBI) (Fig 1).

Products mapping to track changes in competitiveness of the Polish agri-food sector on the EU and US markets and identify export specializations was performed for the years 2004 and 2021. The analysis covered those 2-digit HS items, which share in the structure of bilateral trade flows (exports or imports) in at least one year covered by the study was 5% or more.

## Results and discussion

### Poland’s agri-food trade value and pattern

**Fig 2:**
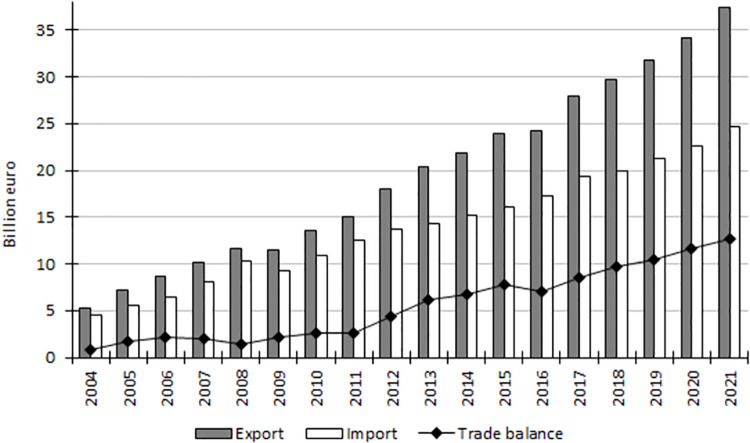
Poland’s foreign trade in agri-food products in 2004–2021 (billion euro). Source: [13], the authors’ elaboration.

Incorporation of Poland into the European Single Market as well as the adoption of the EU acquis communautaire in the trade policy resulted in a marked stimulation of trade in agri-food products. In the years 2004–2021 the value of agri-food export from Poland increased over 7-fold, while that of import grew 5.5-fold, reaching in the last investigated year 37.4 billion euro and 24.7 billion euro, respectively (Fig 2). The dynamic growth in the value of trade resulted first of all from the intensification of trade with other EU member countries thanks to the reduction of trade barriers, while in the initial period of Poland’s EU membership it was also facilitated by a very good preparation of the agri-food industry to operate within the European Single Market and meeting specific sanitary, phytosanitary, veterinary, animal welfare and environmental standards being an obligatory condition to be granted unlimited access to the European Single Market [27].

**Fig 3:**
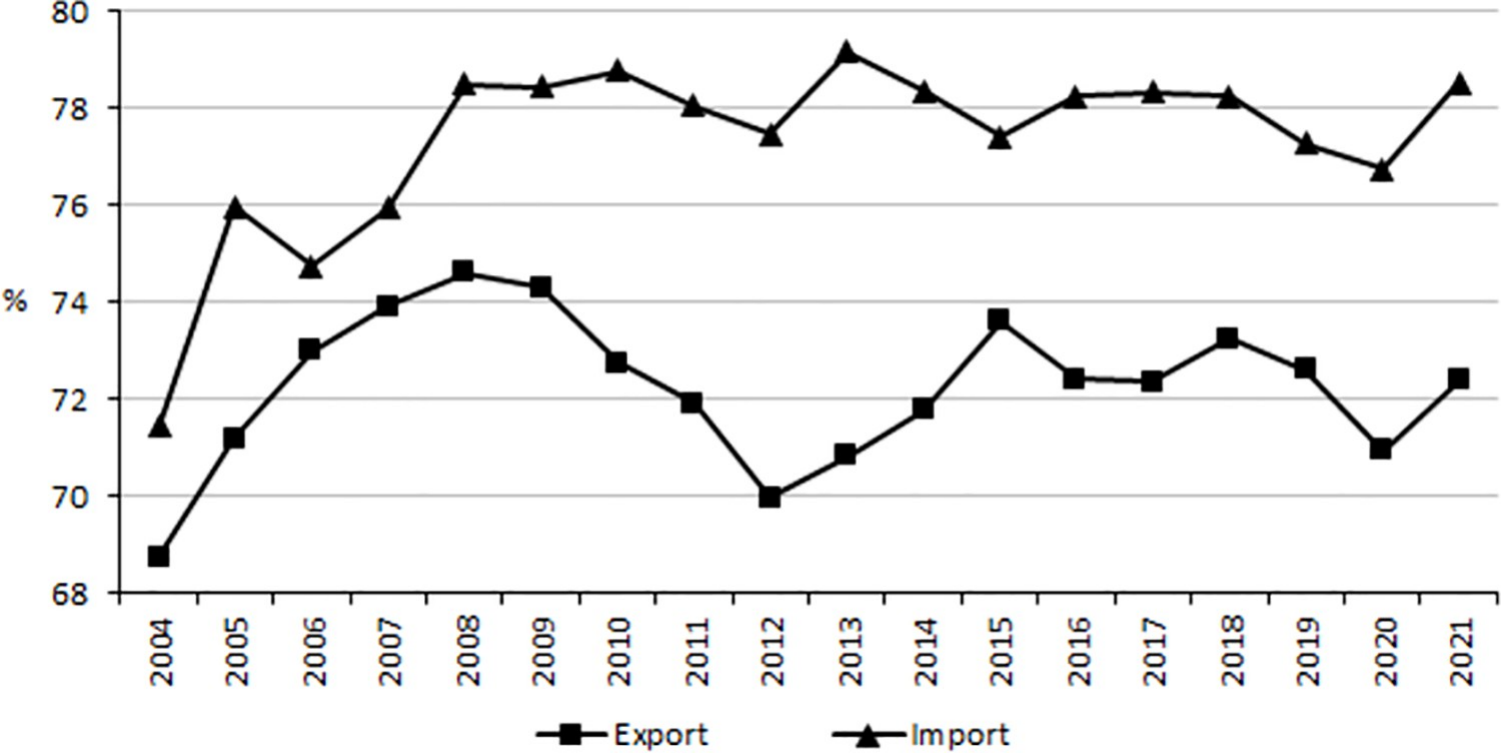
Share of intra-EU trade in agri-food products in total Poland’s trade in agri-food products in 2004–2021 (%). Source: [13], the authors’ elaboration.

Although the trade creation effect was gradually decreasing, the value of agri-food exports from Poland to markets of the other EU member countries increased in the analyzed period almost 7.5-fold, while that of import over 6-fold, leading to an increase in the share of intra-regional trade in total agri-food trade (over the entire analyzed period the values of trade with the EU countries do not include the United Kingdom, which left the EU in 2021). In 2021 the share of the other EU countries in Polish agri-food export was almost 72.5%, while in import it accounted for 78.5%, i.e. it was by 3.6 and 7.1 percentage points more than in 2004 (Fig 3). Together with the creation effect, as a consequence of changes in relative prices a trade diversion was also observed, manifested in the substitution of cheaper imports from more efficient countries outside the customs union with imports from the less efficient, but preferentially treated partner countries.

On the one hand, the observed dynamic growth in trade within the EU showed that tariffs as well as quotas and technical restrictions binding in the pre-accession period constituted barriers for the development of mutual trade relations. On the other hand, this dynamic growth confirmed adequate preparation of food sector enterprises to new competitive conditions. It needs to be remembered here that the dynamic increase in the value of agri-food export following Poland’s accession to the EU to a considerable extent resulted from the cost and price advantages. Further development of export based on this criterion was hindered due to the price convergence in Poland and the other EU countries, as well as increased competition from countries (both EU members and non-EU countries) equipped not only with cost and price advantages (thanks to natural conditions facilitating agricultural production, availability of input resources, lower processor margins or the scale of production), but also technological advantages. To be able to effectively compete with others is was necessary to utilize instruments of non-price competition, either being qualitative or stemming from efficiency advantages determined by the scale of production [36].

It needs to be stressed here that the upward trend for the value of turnover in the agri-food sector was maintained over the entire investigated period, except for 2009, when as a result of economic slowdown in Western Europe domestic demand was decreasing, which–despite weakening of the Polish zloty in relation to euro and the US dollar–led to a decrease in food export from Poland. At the same time depreciation of the Polish zloty compounded by the economic and financial crisis led to a decreased import demand. Due to the subsistence character of supplied goods the agri-food trade quickly overcame problems caused by the crisis and recovered rapidly. Low elasticity of food demand makes agri-food trade more resilient to macro-economic-related exogenous shocks compared to trade in other products and services. This thesis is confirmed by values of indicators for the dynamics of agri-food trade and total Polish trade, recorded both during the 2008–2009 financial crisis, slowdown in the years 2012–2013, and the COVID-19 pandemic in 2020 (Table 1). The latter observation is in line with the study by Szczepaniak et al. [78]. High resilience of the agri-food sector in the face of the COVID-19 pandemic at the global level was also demonstrated by OECD/FAO [79] and Arita et al. [80], while agri-food trade expansion in Hungary was reported by Mizik [46].

**Table 1:** Dynamics of polish foreign trade in 2005–2021 (previous year = 100).

Years	Export		Import	
	All goods	Agri-food products	All goods	Agri-food products
2005	119.2	135.8	113.3	123.9
2006	122.7	119.6	123.8	117.9
2007	115.9	117.7	119.6	124.5
2008	113.3	114.8	117.4	126.3
2009	84.4	98.5	75.5	90.6
2010	123.1	118.0	125.3	118.0
2011	112.5	111.7	112.6	114.5
2012	106.4	119.2	102.4	108.9
2013	107.0	113.0	100.9	104.5
2014	107.4	107.5	107.7	106.1
2015	108.3	109.2	105.2	106.2
2016	102.6	101.6	101.8	107.2
2017	112.6	115.1	114.7	112.3
2018	107.6	106.3	110.1	103.3
2019	106.7	107.1	104.0	106.4
2020	100.4	107.6	96.5	106.4
2021	119.5	109.4	125.3	109.0

Source: [13], the authors’ elaboration.

Changes in trade dynamics were reflected in the balance of trade in agri-food products. In the years 2004–2021 Poland was an exporter of agri-food products, while except for the years 2007–2008 and 2016 the value of trade surplus increased, being a factor balancing the deficit or markedly improving the positive total trade balance. In 2021 the positive balance of trade in the agri-food sector amounted to 12.7 billion euro and it was over 15-fold greater than that in the year of Poland’s accession to the EU (Fig 2). As much as 60% of the value resulted from the surplus recorded in trade with the other EU countries (7.7 billion euro in 2021; Table 2).

**Table 2:** Commodity structure of Poland’s foreign trade in agri-food products with the other EU countries in 2004 and 2021.

Specification	Export				Import				Trade balance	
	2004	2021			2004	2021			2004	2021
	Million euro		%	2004 = 100	Million euro		%	2004 = 100	Million euro	
Live animals; animal products	1 264.7	7 729.9	28.6	611.2	544.8	5 002.7	25.8	918.3	719.9	2 727.2
Vegetable products	870.4	4 377.7	16.2	502.9	1 046.0	5 062.6	26.1	484.0	-175.6	-684.9
Animal or vegetable fats and oils	34.9	712.9	2.6	2 041.5	223.2	1 106.4	5.7	495.6	-188.3	-393.6
Prepared foodstuffs; beverages, spirits and vinegar; tobacco and manufactured tobacco substitutes	1 474.3	14 248.7	52.6	966.5	1 368.0	8 194.0	42.3	599.0	106.2	6 054.8
Total	3 644.3	27 069.2	100.0	742.8	3 182.0	19 365.7	100.0	608.6	462.2	7 703.5

Source: [13], the authors’ elaboration.

**Fig 4:**
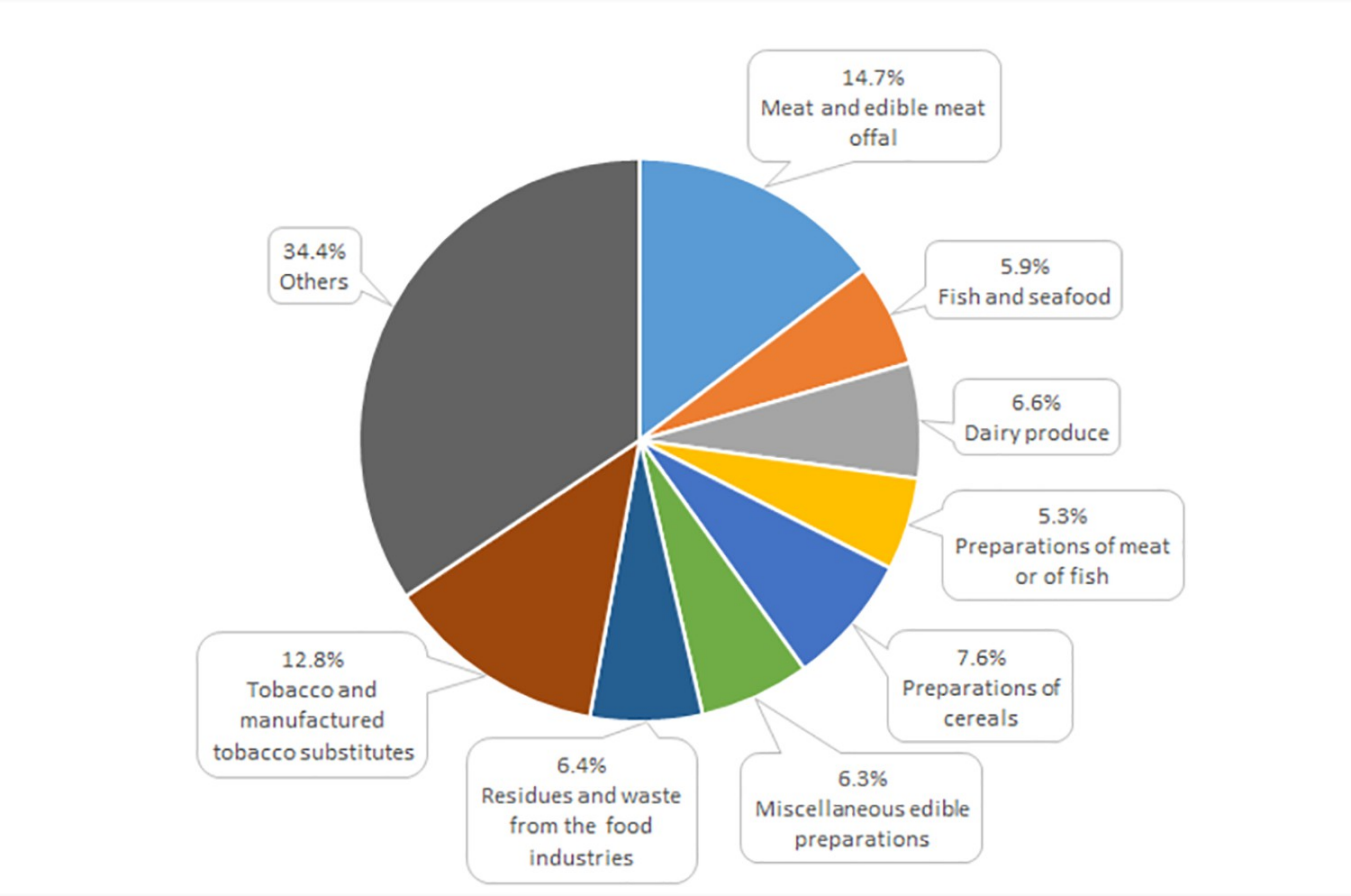
Commodity structure of Poland’s export of agri-food products to the other EU countries in 2021 (%). Source: [13], the authors’ elaboration.

The Polish agri-food trade with the other EU countries was dominated by food preparations and animal origin products, in 2021 accounting for over 80% total export and approximately 70% import (Table 2). In 2021 these two product categories generated jointly the positive balance of trade amounting to 8.8 billion euro, exceeding by 15% surplus in the total intra-EU trade. However, it was reduced by the deficit in trade of plant origin products as well as oils and fats. In 2021 the most important goods in agri-food export from Poland to the other EU countries included meat and offal, tobacco and manufactured tobacco substitutes, preparations of cereals, dairy produce, fish and seafood, residues and waste from the food industries as well as preparations of meat and fish. Jointly these above-mentioned assortment groups provided Poland with 60% total revenue from food export to the European Single Market (Fig 4). Their high share in exports resulted from the modernization processes implemented in food processing plants and reflected adaptation of the Polish export offer to the requirements of consumers from the highly developed EU countries [81]. All the above-mentioned groups of products generated a positive balance of trade, growing in relation to that of 2004 [13].

**Fig 5:**
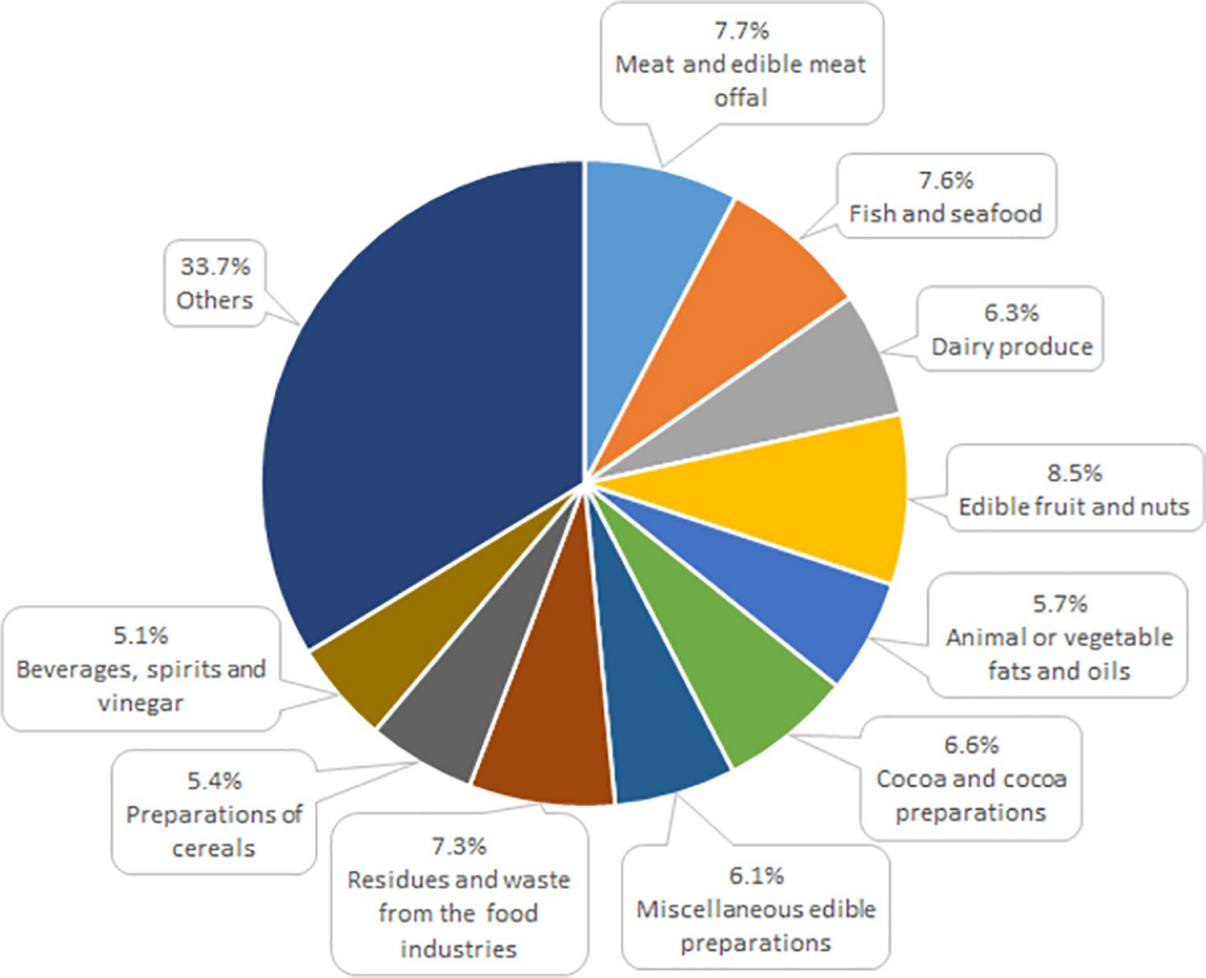
Commodity structure of Poland’s import of agri-food products from the other EU countries in 2021 (%). Source: [13], the authors’ elaboration.

Poland imported primarily fruit and nuts, meat and offal, fish and seafood, residues and waste from the food industries, cocoa and cocoa preparations, dairy produce, oils and fats, preparations of cereals and beverages. In 2021 imports of these assortment groups accounted jointly for 60% expenditure for food imports from the other EU countries, while including various food preparations it was 66% total expenditure (Fig 5). In the years 2004–2021 in trade of fruit and nuts, oils and fats as well as beverages a progressing deficit was observed in the balance of trade, which in the last analyzed year was highest (704 million euro) in trade of horticultural products [13]. This resulted predominantly from the required import of fresh fruit grown in warmer climatic zones or grown in Poland only seasonally. However, it needs to be stressed that due to the definitely complementary character of the fruit species structure of export and import, the recorded trade deficit in this respect needs to be considered a positive phenomenon, indicating an extension of the market offering and more effective satisfaction of consumer demand from the population with a growing purchasing power. A negative balance of trade in oils and fats as well as beverages may be perceived analogously [24]. Among oil crops only rape is grown and processed on a large scale in Poland, thus demand for oils manufactured from other oil crops (e.g. palm, sunflower or coconut oils, dehydrogenated vegetable oils, oils for industrial uses) is met mostly through import. In turn, beverage imports include primarily malt beer, sparkling wines, vermouths and other wines produced in the EU, cider and undenatured ethyl alcohol of an alcoholic strength over 80% [13].

Trade with non-EU countries accounted for approximately 30% Polish export and 20% import of agri-food products. In 2004 the USA ranked third and in 2021 the fourth export partner in this group of countries (Table 3). In terms of the value of export the United Kingdom (in accordance with the present-day status the United Kingdom is treated as a non-EU country), Ukraine and Russia were more important target markets. In view of the geographical proximity of the British market, the purchasing power of British consumers as well as advantageous trade conditions (after Brexit trade between the United Kingdom and the EU countries continues to be duty-free [82]) the position of United Kingdom in the structure of Polish agri-food trade seems secure. In view of the on-going military conflict between Russia and Ukraine the non-market risk for bilateral trade relations between Poland and the two countries, being high previously, is currently further increasing particularly in trade with Russia. Another problem is the change in effective demand for Polish products on both markets determined by the conflict. In this context the importance of the USA as a partner in food export may be relatively increasing.

**Table 3:** Major trading partners in Poland’s agri-food trade in 2004 and 2021.

No.	Export (billion euro)				Import (billion euro)			
	Country	2004	Country	2021	Country	2004	Country	2021
1	Germany	1 359	Germany	9 389	Germany	820	Netherlands	2 876
2	Russia	409	United Kingdom	2 973	Netherlands	565	Spain	1 357
3	Netherlands	317	Netherlands	2 240	Spain	303	Belgium	1 292
4	United Kingdom	307	France	2 110	France	224	Italy	1 276
5	Czechia	283	Italy	1 903	Italy	218	France	1 109
6	Italy	277	Czechia	1 593	Argentina	212	Denmark	1 000
7	France	178	Spain	1 085	Denmark	195	Sweden	937
8	Hungary	170	Romania	948	Czechia	167	Ukraine	918
9	United States	162	Belgium	928	Belgium	156	Czechia	783
10	Denmark	145	Hungary	921	Hungary	141	Argentina	678
11	Ukraine	142	Slovakia	844	China	134	Hungary	467
12	Lithuania	133	Ukraine	812	Norway	89	Slovakia	432
13	Belgium	124	Denmark	770	Brazil	84	Brazil	413
14	Slovakia	98	Lithuania	713	Sweden	77	Lithuania	411
15	Belarus	96	Russia	676	United Kingdom	76	Norway	393
16	Sweden	86	Sweden	646	Greece	66	Austria	389
17	Austria	79	United States	612	Slovakia	64	China	276
18	Spain	76	Austria	601	Ecuador	56	United Kingdom	268
19	Romania	68	Saudi Arabia	511	United States	53	Ireland	261
20	Latvia	61	Algeria	418	Austria	50	Romania	247
21	Croatia	36	Ireland	332	Ukraine	48	Russia	240
22	Switzerland	35	Latvia	327	Ivory Coast	48	Greece	210
23	Algeria	35	Bulgaria	312	Turkey	46	Turkey	197
24	Estonia	34	Greece	304	Viet Nam	42	United States	175
25	Bosnia & Herzegovina	32	Israel	293	Bulgaria	32	Paraguay	157

Source: [13], the authors’ elaboration.

**Fig 6:**
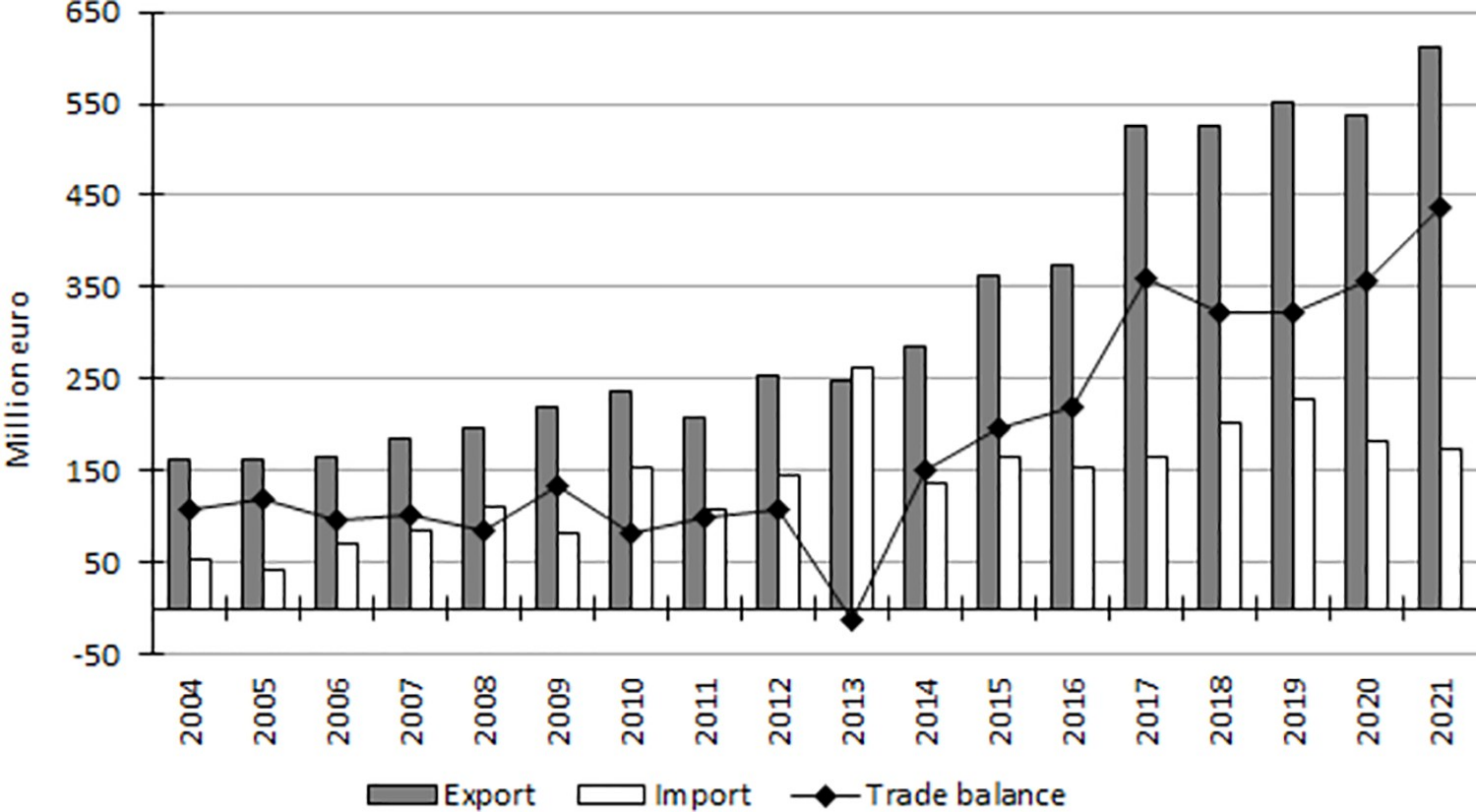
Poland’s foreign trade in agri-food products with the USA in 2004–2021 (million euro). Source: [13], the authors’ elaboration.

In the years 2004–2021 the value of agri-food export from Poland to the USA increased almost 4-fold, in the last of the analyzed years reaching 612 million euro (Fig 6). A slightly less dynamic increase was recorded for food import, which in 2021 amounted to 175 million euro. It needs to be stressed here that over the entire analyzed period, except for 2013, Poland recorded a surplus in its agri-food trade with the USA, since 2017 amounting to 324–437 million euro (Fig 6). What is essential, the positive trade balance was recorded both in trade of agricultural commodities and products of the food industry, being the greatest (308.7 million euro) in trade of prepared foodstuffs, beverages and tobacco products, which accounted for almost 65% and 46% total export and import of agri-food products to and from the USA, respectively (Table 4).

**Table 4:** Commodity structure of Poland’s foreign trade in agri-food products with the USA in 2004 and 2021.

Specification	Export				Import				Trade balance	
	2004	2021			2004	2021			2004	2021
	Million euro		%	2004 = 100	Million euro		%	2004 = 100	Million euro	
Live animals; animal products	23.4	137.3	22.4	585.7	14.5	53.7	30.7	370.9	9.0	83.6
Vegetable products	21.0	83.5	13.6	397.8	16.7	40.3	23.0	240.6	4.2	43.2
Animal or vegetable fats and oils	0.6	2.3	0.4	374.8	1.4	0.7	0.4	50.0	-0.8	1.6
Prepared foodstuffs; beverages, spirits and vinegar; tobacco and manufactured tobacco substitutes	116.8	388.8	63.5	333.0	20.8	80.2	45.9	385.0	96.0	308.7
Total	161.8	611.9	100.0	378.1	53.5	174.8	100.0	327.0	108.3	437.1

Source: [13], the authors’ elaboration.

**Fig 7:**
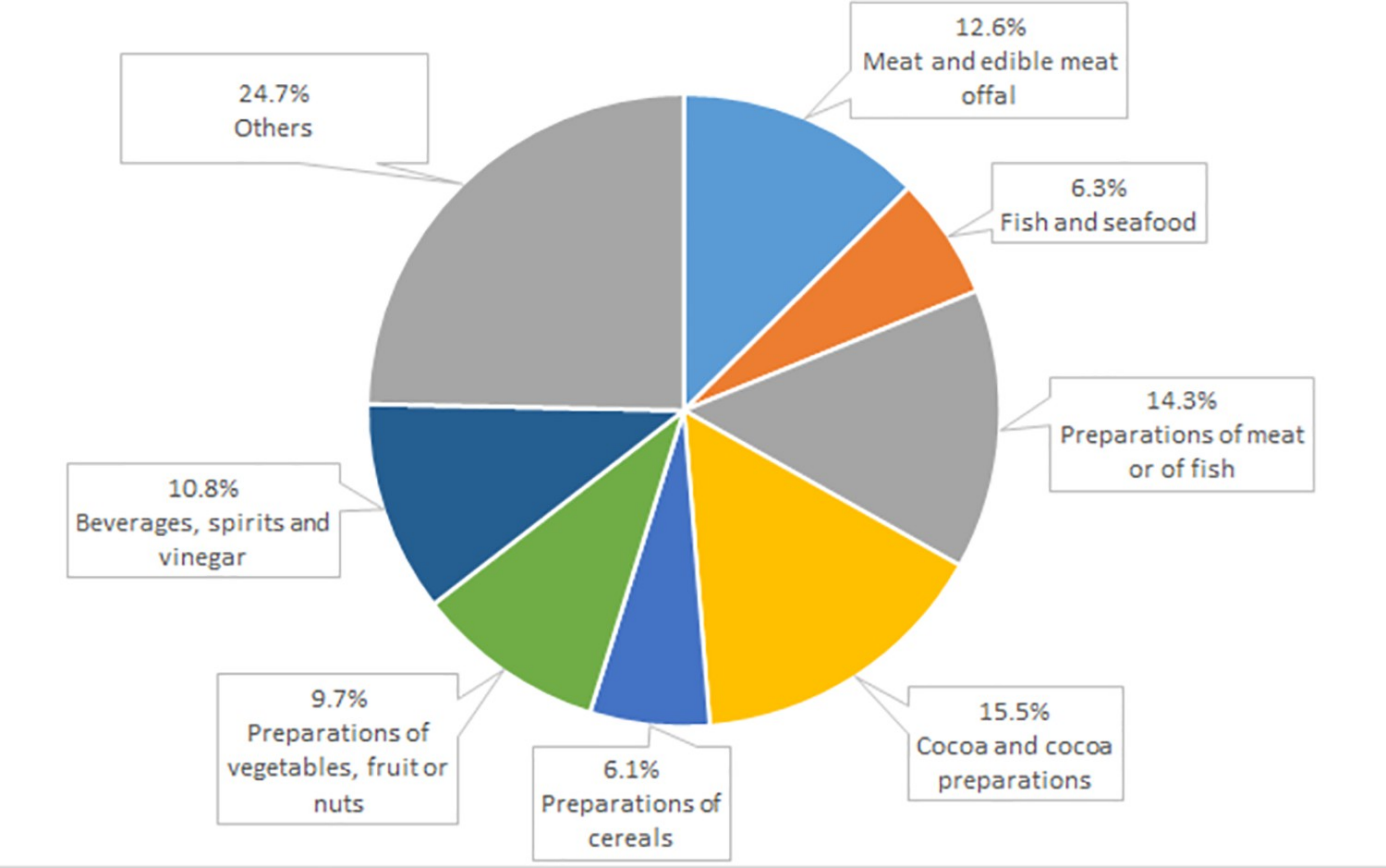
Commodity structure of Poland’s export of agri-food products to the US in 2021 (%). Source: [13], the authors’ elaboration.

Among the prepared foodstuffs the most important exported items in the structure of exports to the USA included cocoa and cocoa preparations (15.5% total export in 2021), preparations of meat and fish (14.3%; while the USA is self-sufficient in terms of meat production, the US fishing industry does not satisfy the domestic demand, as a consequence stimulating import), non-alcoholic and alcoholic beverages (10.8%) as well as preparations of vegetables, fruit and nuts (9.7%), in this case primarily fruit juices (Fig 7). Moreover, in 2021 over 12.5% total revenue from agri-food export to the USA (76.9 million euro) was provided by meat and offal. Jointly the above-mentioned groups of products generated 80% total surplus in the agri-food trade of Poland with the USA.

**Fig 8:**
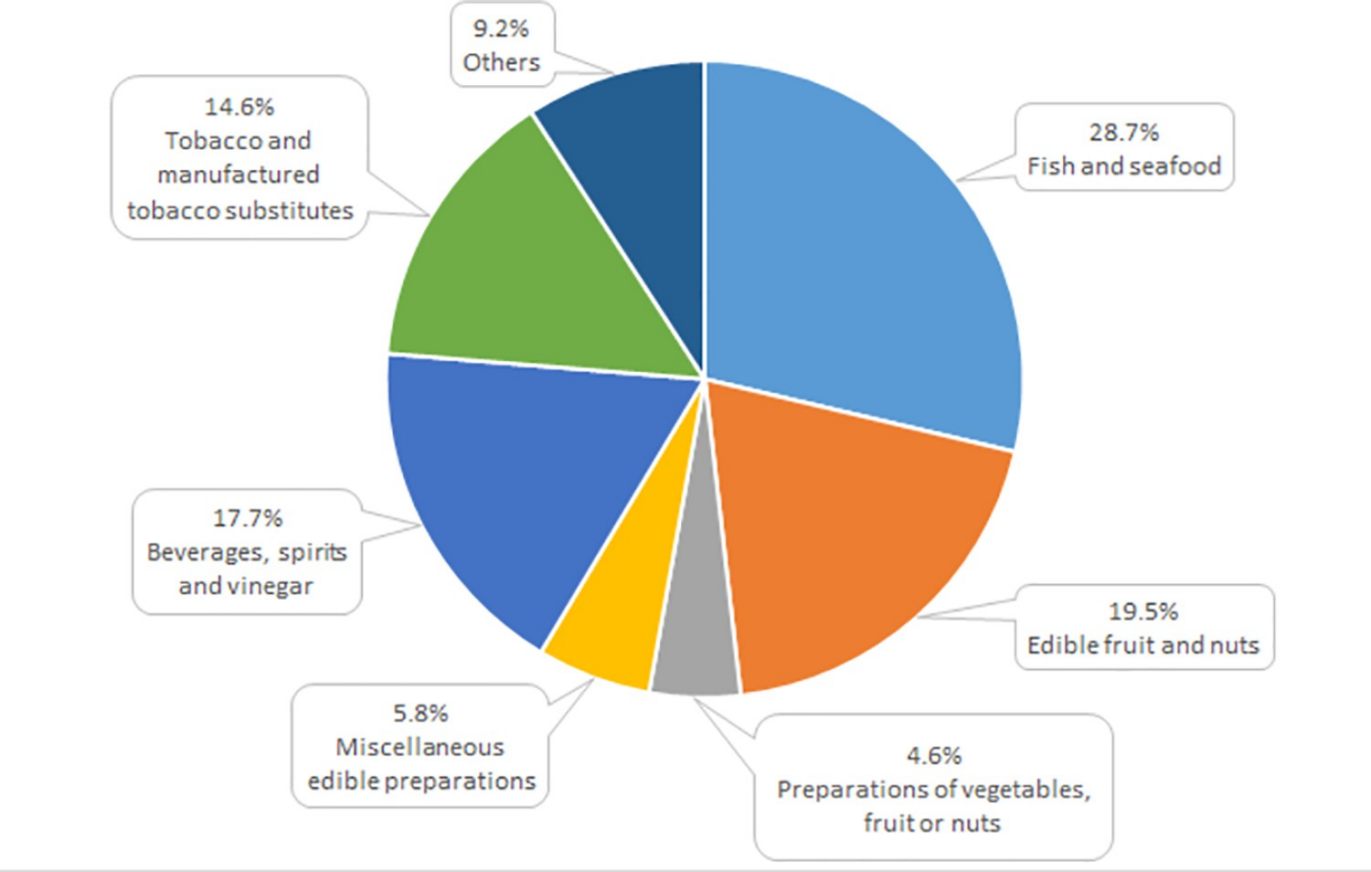
Commodity structure of Poland’s import of agri-food products from the US in 2021 (%). Source: [13], the authors’ elaboration.

Three groups of products had a negative effect on the balance of bilateral trade, in 2021 accounting for almost 65% total expenditure incurred for food import from the USA, while together with beverages it was as much as 80%. The above-mentioned groups were fish, crustaceans and molluscs (28.7% total import in 2021), fruit and nuts (19.5%) as well as tobacco and manufactured tobacco substitutes (14.6%; Fig 8). In 2021 the trade deficit in the case of these product groups amounted to almost 12 million euro, 28 million euro and over 25 million euro, respectively, which jointly reduced Poland’s profits from agri-food trade with the USA by approximately 15% (Table 4). However, it needs to be stressed here that in terms of product availability on the market the import was largely necessary. Imports to Poland included first of all fruits from other climate zones, nuts, fish and seafood as well as wines and whisky, which either are not produced in Poland at all or which production volume is too low to meet the demand. While in the case of products complementary or supplementary to the domestic production deficit increased, import does not constitute a direct threat for domestic producers. In the case of tobacco and tobacco products it enhances the competitive pressure on tobacco farmers, which meet the demand of the domestic market with a surplus.

Analysis of the assortment structure in agri-food trade of Poland with the USA indicates that the American market, at the relatively limited importance for the sector in the macro scale, is essential for several branches of the Polish food industry, including e.g. the meat, fish, confectionery, spirit, tobacco and fruit processing industries. What is crucial, these are the branches of the Polish food industry which are characterized by the greatest degree of globalization measured by the share of transnational corporations in the revenue of this sector and in the value of production of the food industry [83].

### Distribution of comparative advantages of the Polish agri-food sector

The comparative advantage theory postulates that in the case of free trade and ideally competitive markets trade flows result from differences in production capacities, determined by the diverse resources of production factors in individual countries and that a country will specialize in the production of this good, for which it has a relative cost advantage over its partners [45,84]. When analyzing values of the Balassa, Vollrath and Revealed Symmetric Comparative Advantage indices it may be stated that in 2004 Poland had revealed comparative advantages (RCA>1, RC>0, 0<RSCA<1) in export to the European Single Market for 8 out of 15 groups of products crucial in the structure of bilateral trade (a >5% share in export or in import in minimum one year covered by the analysis) (Table 5). These were animal origin products (HS 02, HS 03, HS 04, HS 16), fresh vegetables (HS 07) as well as preparations of vegetables and fruit (HS 20), sugar confectionary (HS 17) and preparations of cereals (HS 19). The most advantageous competitive situation was observed in the intra-EU export of dairy produce (HS 04), as well as preparations of meat and of fish (HS 16). What is important, preparations of meat and of fish next to meat and offal (HS 02), tobacco and manufactured tobacco substitutes (HS 24) were the only of the above-mentioned assortment groups, for which in the period up to 2021 comparative advantages obtained in the European Single Market increased. This indicates the strengthening of the competitive capacity on the EU market of meat and processed meat products as well as reaching such a capacity in the trade of tobacco and its substitutes.

**Table 5:** Comparative advantages of the polish agri-food sector in trade with the other EU countries in 2004 and 2021.

Specification	HS code	2004				2021			
		RCA	RC	RSCA	TBI	RCA	RC	RSCA	TBI
Meat and edible meat offal	02	1.68	1.04	0.26	0.32	1.89	1.28	0.31	0.45
Fish and crustaceans, molluscs and other aquatic invertebrates	03	1.23	0.42	0.10	0.17	0.78	-0.49	-0.12	0.04
Dairy produce	04	5.60	3.44	0.70	0.73	1.06	0.11	0.03	0.19
Edible vegetables	07	2.09	1.47	0.35	0.41	0.68	-0.77	-0.19	-0.02
Edible fruit and nuts	08	0.74	-0.61	-0.15	-0.08	0.41	-1.78	-0.42	-0.27
Animal or vegetable fats and oils	15	0.14	-3.98	-0.76	-0.73	0.46	-1.55	-0.37	-0.22
Preparations of meat, of fish or of crustaceans, molluscs or other aquatic invertebrates	16	3.50	2.50	0.56	0.60	3.09	2.26	0.51	0.62
Sugars and sugar confectionery	17	1.93	1.31	0.32	0.38	0.89	-0.23	-0.06	0.11
Cocoa and cocoa preparations	18	0.82	-0.41	-0.10	-0.03	0.74	-0.60	-0.15	0.02
Preparations of cereals	19	1.46	0.76	0.19	0.25	1.40	0.67	0.17	0.32
Preparations of vegetables, fruit or nuts	20	2.30	1.67	0.39	0.45	1.03	0.05	0.01	0.18
Miscellaneous edible preparations	21	0.63	-0.93	-0.23	-0.16	1.05	0.09	0.02	0.19
Beverages, spirits and vinegar	22	0.46	-1.57	-0.37	-0.31	0.62	-0.94	-0.23	-0.07
Residues and waste from the food industries; prepared animal fodder	23	0.23	-2.97	-0.63	-0.59	0.88	-0.25	-0.06	0.11
Tobacco and manufactured tobacco substitutes	24	0.45	-1.62	-0.38	-0.32	3.31	2.39	0.54	0.64

Source: [13], the authors’ elaboration.

Those observations are in line with findings by Bojnec and Ferto [28,29] and Pawlak [36], who noticed that after the EU enlargement towards Central and Eastern European countries the revealed comparative advantages gained by the new EU member states increased. However, at the same time the competitive pressure increased, which caused difficulties with maintaining the strong competitive position in the long run. Among the other Central European countries Poland experienced a relatively high duration of trade advantages in differentiated products [29], including the above-mentioned meat preparations and tobacco products. Competitiveness of the Polish meat industry resulted from the considerable production potential of farms, a high standard of modernization in processing plants capable of providing products satisfying consumer requirements on markets of western European countries, as well as cost and price advantages thanks to lower labor costs and processors’ margins rather than lower raw meat materials [24,81]. Factors determining the competitive position of the Polish tobacco industry in the EU include the production potential, compliance of the raw materials with strict quality standards concerning e.g. adequate moisture content, leaf structure and color or nicotine content, as well as price advantages both at the raw material production and processing levels [85]. It needs to be stated here that the development of production and improvement of tobacco quality were promoted by the support for tobacco growers within the EU CAP, whereas the potential of the tobacco industry enterprises, which in the first years after Poland’s accession to the EU were characterized by an average level of capital assets per employee and efficiency of input utilization [86], developed thanks to the inflow of foreign capital in the form of foreign direct investments. These contributed to an improvement of the technical and technological standard of plants producing tobacco and tobacco products as well as development of sales and export [87].

In contrast to meat and tobacco products, despite an increased value of export a cause for concern is observed in the weakening of Poland’s competitive position in the intraregional trade in the other groups of products, leading e.g. to the absolute loss of advantages in export of vegetables. The decreasing comparative advantages or in extreme cases even their absence may not necessarily be interpreted as a negative situation in relation to the groups of products, which are simultaneously subject to necessary import and are connected with intra-industry trade (e.g. fresh vegetables, fruit, oil seeds and their processed products) [25]. However, this situation needs to be considered a threat to branches characterized by a high production potential exceeding the needs of the domestic market and those based on domestic raw materials, such as e.g. the dairy, sugar and fruit and vegetable processing industries. Weakening of Poland’s competitive position in these branches resulted among other things from the gradual loss of cost and price advantages experienced in the first period of EU membership, resulting from low labor costs and limited consumption of chemicals [24,81]. Despite the progressing concentration processes in production and processing, problems were also caused by the considerable farm fragmentation [68] and the scattered distribution of processing plants resulting in labor productivity being lower than in the EU-15 and the USA [88].

**Fig 9:**
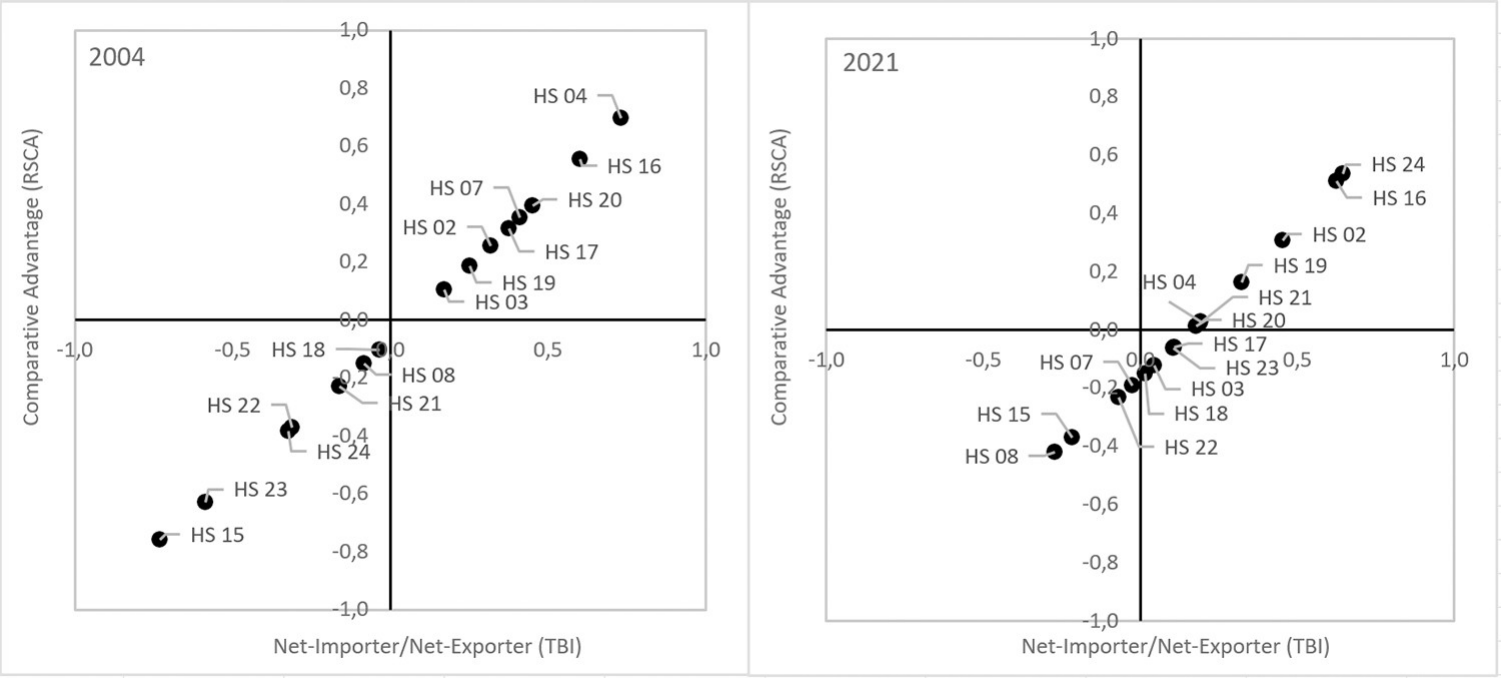
Products mapping for selected agri-food product groups exported from Poland to the other EU countries in 2004 and 2021 (Widodo’s method). Source: [13], the authors’ elaboration.

Ranking of products depending on their level of comparative advantages (i.e. Revealed Symmetric Comparative Advantages–RSCA) and the degree of their export specialization (in terms of the Trade Balance Index–TBI) showed that in both analyzed years the groups of products, in the trade of which Poland reached high comparative advantages and positive balance of trade (group A) accounted for approximately 60% total value of export of agri-food products to the other EU countries (Fig 9, Table 6). These included meat and offal (HS 02), dairy produce (HS 04), preparations of meat and of fish (HS 16), preparations of cereals (HS 19), preparations of vegetables and fruit (HS 20), in 2004 it was fish (HS 03), while in 2021 tobacco and manufactured tobacco substitutes (HS 24). It needs to be stressed that despite the reduction in the share of these assortment groups in the total export to the European Single Market by 4.5 percentage points, Poland strengthened its position as their net exporter. Trade surplus generated by trade in these products amounted to 1.3 billion euro in 2004 and 8.8 billion euro in 2021, i.e. almost 3-fold and by 15% more than the positive total balance of trade with the EU countries (Table 2). When looking at the export shares, among those most competitive products the highest export potential was found for poultry meat and edible offal (HS 0207), cheese and curd (HS 0406), prepared or preserved poultry meat (HS 1602) and fish (HS 1604), bread, pastry, cakes and biscuits (HS 1905), apple juice and other fruit juices (HS 2009) and cigarettes (HS 2402) [13].

**Table 6:** Shares and trade balance of individual product groups resulting from Widodo’s matrix in the total value of agri-food trade between Poland and the other EU countries.

Item	2004			2021		
	Share in the total value of export (%)	Share in the total value of import (%)	Trade balance (million euro)	Share in the total value of export (%)	Share in the total value of import (%)	Trade balance (million euro)
Group A	62.0	29.7	1 313.1	57.5	35.2	8 757.2
Group B	x	x	x	x	x	x
Group C	x	x	x	19.1	23.6	608.5
Group D	25.4	49.8	-661.7	12.7	24.3	-1 270.7

Notes: Shares do not jointly amount to 100%, since this analysis includes only these groups of products, which share in the structure of export or import in minimum one year covered by these investigations was min. 5%.

Source: [13], the authors’ elaboration.

In 2021 Poland was also a net exporter of fish (HS 03), sugar and sugar confectionery (HS 17), cocoa and coca preparations (HS 18) as well as residues and waste from the food industry (HS 23), although in their trade it had no comparative advantages (group C). These products accounted for almost 20% in the structure of agri-food export from Poland to the other EU countries and raised trade surplus by 608.5 million euro. Smoked fish fit for human consumption (HS 0305), sugar confectionery not containing cocoa (HS 1704), white sugar and other sugars (HS 1701), chocolate and other food preparations containing cocoa (HS 1806), as well as preparations used in animal feeding (HS 2309) represented the highest export shares in the European Single Market [13].

The assortment groups, in the trade of which the absence of comparative advantages was accompanied by a lack of export specialization and a negative trade balance (group D), in 2004 and 2021 accounted for 25% and 13% export, respectively, while they were responsible for almost 50% and 25% total expenditure for food import from the other EU countries. Trade deficit observed in the analyzed period in trade of these products doubled, in 2021 reaching 1.3 billion euro, which reduced the positive total balance of trade by over 16%. This group of products included fruit (HS 08), oils and fats (HS 15), beverages and spirits (HS 22), in 2004 cocoa and cocoa preparations (HS 18), residues and waste from the food industry (HS 23) as well as tobacco and manufactured tobacco substitutes (HS 24), while in 2021 it was vegetables (HS 07). Many of those products were subject to intra-industry trade. A very specific intra-industry nature of trade with the other EU member states was made in fruit and vegetables. For instance, in 2021 fresh mushrooms (HS 0709), fresh strawberries and other soft fruits (HS 0810), as well as raspberries and other frozen fruits (HS 0811) were exported from Poland, while the most imported products included fresh tomatoes (HS 0702), fresh bananas (HS 0803), fresh or dried avocados (HS 0804), citrus fruits (HS 0805) and grapes (HS 0806). A similar situation was observed in trade in beverages. Being an exporter of waters (HS 2201), beer made from malt (HS 2203) and undenatured ethyl alcohol (HS 2207) Poland imported from the other EU countries mainly wines (HS 2204) [13].

It may be observed here that in the period following its accession to the EU Poland maintained its strong and relatively stable competitive position in the intraregional trade in animal origin products as well as preparations of cereals, fruit and vegetables, while it considerably enhanced its advantages in trade of tobacco products. They are traditional export specialties of Poland, created based on the production potential of the agricultural sector, quality agricultural raw materials, resulting among other things from the relatively limited use of agrochemicals, lower wages and processing margins as well as the diverse range of food products, meeting commercial quality standards binding in markets of developed countries [24]. In turn, Poland took a disadvantageous competitive position in trade of products, which were important imported goods and for various reasons their import was necessary (fresh fruit and vegetables, oils and vegetable fats, beverages). In terms of Ricardo’s theory of comparative advantage and the theory of factor endowments in the Heckscher-Ohlin-Samuelson model it may be stated that comparative advantages attained by Poland in the European Single Market were sources of advantageous export specialization, while the structure of trade developed on their basis was coherent. However, the weakening of the competitive position of the Polish agri-food sector recorded in trade of most key products resulted partly from the decreasing cost and price advantages and partly from the degree of saturation of the European Single Market and the limited potential to increase the EU food demand may be a cause for concern. In this context when searching for the possibilities to expand sales to non-EU markets the competitiveness of agri-food products exported from Poland to the US market was assessed.

**Fig 10:**
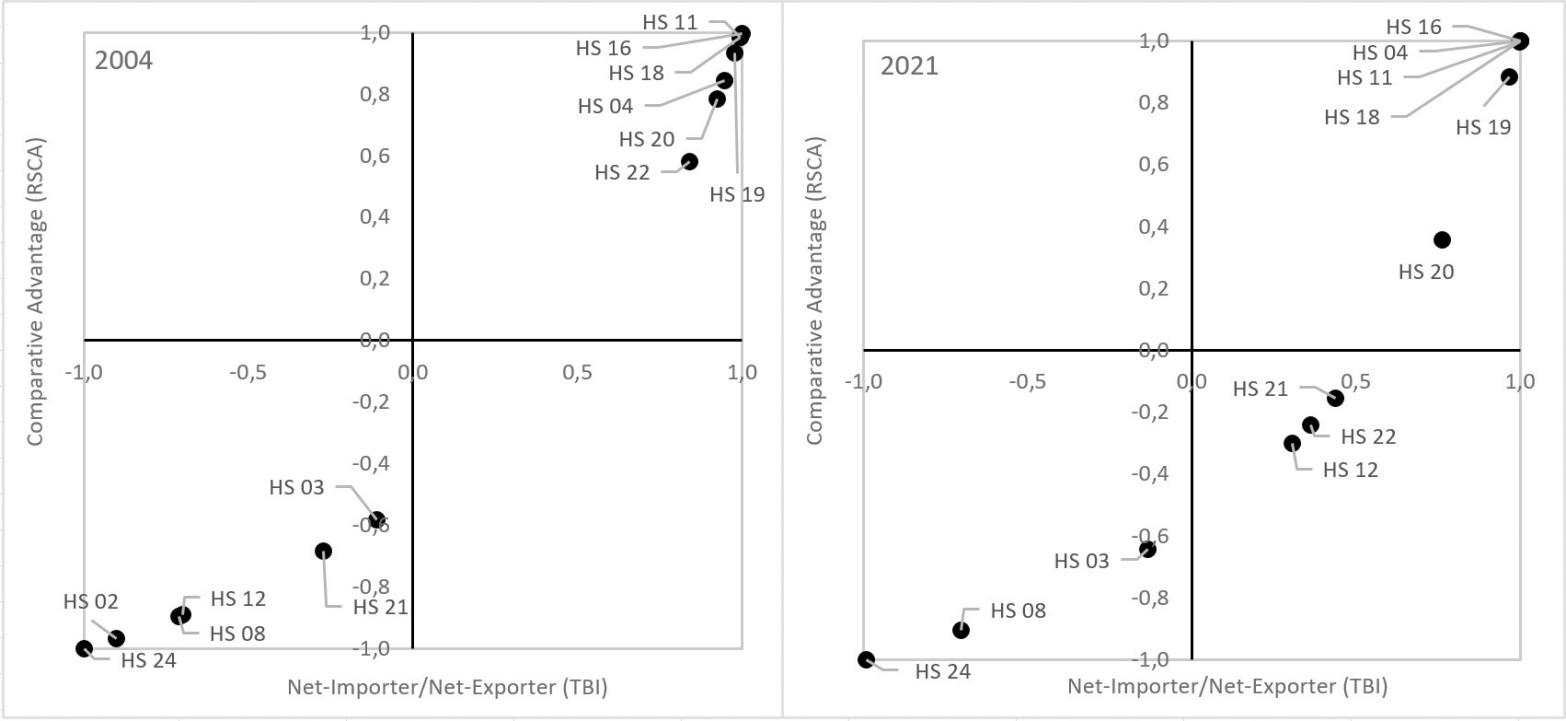
Products mapping for selected agri-food product groups exported from Poland to the USA in 2004 and 2021 (Widodo’s method). Source: [13], the authors’ elaboration.

Poland attained revealed comparative advantages (RCA>1, RC>0, 0<RSCA<1) in export to the US market in the case of all basic foodstuffs occupying key positions in the structure of exports, i.e. preparations of meat or of fish (HS 16), of cereals (HS 19), of vegetables and fruit (HS 20), cocoa and cocoa preparations (HS 18) as well as dairy produce (HS 04) and products of the milling industry (HS 11) characterized by a lesser processing degree (Table 7, Fig 10). What is essential here, in the years 2004–2021 in export of all the above-mentioned groups of products, except for preparations of vegetables and fruit, the comparative advantages increased and Poland strengthened its net exporter position. It results from the positioning of products according to Widodo that this highly competitive assortment group providing an increasing surplus of trade (group A) in 2004 accounted for almost 82% (together with beverages and spirits–HS 22), while in 2021 50% total export of agri-food products to the USA. The value of the positive trade balance generated in trade of these groups of products amounted to 127.5 million euro and 293.5 million euro, respectively (Table 8). In 2004 thus attained trade surplus by almost 20% exceeded the positive total bilateral trade balance, while in 2021 it determined this balance in approximately 70% (Table 4, Table 8). According to the export shares, the most competitive products with the highest export potential were cheese and curds (HS 0406), potato starch (HS 1108) and wheat gluten (HS 1109), prepared or preserved meat of swine (HS 1602), sardines and other prepared or preserved fish (HS 1604), chocolate and other food preparations containing cocoa (HS 1806), waffles, wafers and other cakes and biscuits (HS 1905), mushroom and other prepared or preserved vegetables (HS 2001), jams and marmalades (HS 2007) and fruit juices (HS 2009) [13].

**Table 7:** Comparative advantages of Polish agri-food sector in trade with the USA in 2004 and 2021.

Specification	HS code	2004				2021			
		RCA	RC	RSCA	TBI	RCA	RC	RSCA	TBI
Meat and edible meat offal	HS 02	0.02	-8.13	-0.97	-0.90	x	x	x	x
Fish and crustaceans, molluscs and other aquatic invertebrates	HS 03	0.27	-2.66	-0.58	-0.11	0.22	-3.04	-0.64	-0.13
Dairy produce	HS 04	11.83	4.94	0.84	0.95	3 276.95	16.19	1.00	1.00
Edible fruit and nuts	HS 08	0.06	-5.78	-0.89	-0.71	0.05	-5.99	-0.90	-0.70
Products of the milling industry	HS 11	119.06	9.56	0.98	0.99	652.08	12.96	1.00	1.00
Oil seeds and oleaginous fruits	HS 12	0.06	-5.69	-0.89	-0.70	0.54	-1.24	-0.30	0.31
Preparations of meat, of fish or of crustaceans, molluscs or other aquatic invertebrates	HS 16	498.52	12.42	1.00	1.00	53 695.93	21.78	1.00	1.00
Cocoa and cocoa preparations	HS 18	123.40	9.63	0.98	0.99	1 590.52	14.74	1.00	1.00
Preparations of cereals	HS 19	29.04	6.74	0.93	0.98	16.44	5.60	0.89	0.97
Preparations of vegetables, fruit or nuts	HS 20	8.21	4.21	0.78	0.92	2.11	1.49	0.36	0.76
Miscellaneous edible preparations	HS 21	0.19	-3.34	-0.68	-0.27	0.73	-0.63	-0.16	0.44
Beverages, spirits and vinegar	HS 22	3.78	2.66	0.58	0.84	0.61	-0.99	-0.24	0.36
Tobacco and manufactured tobacco substitutes	HS 24	0.00	-18.63	-1.00	-1.00	0.00	-14.05	-1.00	-0.99

Source: [13], the authors’ elaboration.

**Table 8:** Shares and trade balance of individual product groups resulting from Widodo’s matrix in the total value of agri-food trade between Poland and the USA.

Item	2004			2021		
	Share in the total value of export (%)	Share in the total value of import (%)	Trade balance (million euro)	Share in the total value of export (%)	Share in the total value of import (%)	Trade balance (million euro)
Group A	81.6	8.4	127.5	49.4	5.0	293.5
Group B	x	x	x	x	x	x
Group C	x	x	x	16.0	25.3	53.7
Group D	11.3	74.3	-21.5	1.0	62.8	-103.7

Notes: Shares do not jointly amount to 100%, since this analysis includes only these groups of products, which share in the structure of export or import in minimum one year covered by these investigations was min. 5%

Source: [13], the authors’ elaboration.

High comparative advantages in export to the USA were observed primarily for easily transported, highly processed products, obtained based on the raw materials, of which Poland has a considerable production potential and a high degree of food self-sufficiency exceeding that of the USA [89]. Competitiveness of the plant origin products exported from Poland is strengthened by their consumer and utility attributes as well as positive opinions on their wholesomeness [24]. Apart from increasing export of processed pork products the US market is seen as showing potential for the export of organic food. Such a scenario could be promoted by reduced tariffs, which in import of fruit and vegetable preparations from Poland, similarly as in the import of dairy and confectionary produce, are relatively high [90]. An additional obstacle is also caused by the presently binding sanitary and phytosanitary and technological barriers, which in the case of the three above-mentioned products are applied more extensively by the USA compared to the EU [91]. As stated by Pawlak [35], the level of comparative advantages in the export of agri-food products from Poland to the USA is closely related to the level of protection of the American market as well as changes in price relations resulting from fluctuations in the exchange rate of the Polish zloty to the US dollar.

The competitive position of Poland improved also in export of oil seeds (HS 12) and various food preparations (HS 21), which in 2021, together with beverages and spirits (HS 22) losing their comparative advantages, despite the relatively disadvantageous situation in relation to the trade partner, provided Poland with the position of a net exporter (group C). Deterioration of the competitive position in trade of beverages resulted from the markedly more dynamic growth of import rather than export in this HS item (Table 4). In 2021 these 3 commodity groups (group C) were responsible jointly for 16% export and 25% total bilateral import of agri-food products to Poland from the USA, generating trade surplus of 53.7 million euro (Table 8). Products comprising this category, characterized by the highest share in export to the US market, included oilseed plants used in perfumery or pharmacy (HS 1211), sauce and preparations thereof (HS 2103), waters (HS 2201), beer made from malt (HS 2203), vodkas and other spirituous beverages (HS 2208) [13].

Products, in the case of which a permanent lack of comparative advantages hindered export specialization and their trade was accompanied by trade deficit (group D) included fish and seafood (HS 03), fruit (HS 08) as well as tobacco and manufactured tobacco substitutes (HS 24). In both analyzed years they were primarily imported–in 2004 they accounted for almost 75%, while in 2021 63% total expenditure for food import from the USA. The value of import for these groups of products exceeded the value of their export by 21.5 million euro and 103.7 million euro, while the trade deficit they created reduced by approximately 20% the value of the positive total trade balance. The most imported products without comparative advantages included frozen salmon and other frozen fish (HS 0304), almonds, pistachios and other nuts (HS 0802), dried prunes (HS 0813) and unmanufactured tobacco (HS 2401) [13].

It results from the conducted analyses that in the years 2004–2021 Poland obtained the greatest comparative advantages, thus improving the positive trade balance in trade with the USA in the case of products of lesser or greater degree of processing, based on domestic raw materials, for which Poland shows a high degree of food self-sufficiency. In accordance with the classical principle of comparative costs, import from the USA consisted of products having no such advantages, to a considerable degree complementary in relation to domestic production, which import was necessary, as a result definitely excluding a negative assessment of lack of competitiveness in a given assortment range.

When observing changes in comparative advantages in trade relations of Poland with the other EU countries and the USA it may be stated that while in intra-regional trade the competitive position of the Polish agri-food sector was weakened, it strengthened on the US market. In view of the limited potential for an increase in food demand in the EU and searching for the potential geographical diversification of markets, the improvement of competitive capacity on the US market needs to be considered advantageous and expected in the long-term perspective. Obviously development of bilateral trade of Poland with the USA would benefit from progressing regulatory cooperation between the EU and the USA, unification of fiscal measures and excise duties, establishment of transparent harmonization rules and mutual recognition of standards as well as an agreement on the protection of EU products with geographical indications by the USA. However, experience of the suspended TTIP negotiations indicates that reaching a compromise in this respect is particularly difficult [19]. In relation to the results of analyses simulating the establishment of a free trade area between the EU and the USA and the possible 80% increase in export of processed food from the EU, including e.g. preparations of cereals and of vegetables and fruit [21] it may be assumed that the competitive position of the EU, including Poland, on the US markets of these products might be strengthened. In this context it seems justified to assume the maintenance of the current export specialization by Poland. However, it needs to be remembered that the potential to gain benefits from preferential trade conditions and development of export onto the US market would require from producers the ability to cope with the competition from specialized, large US farms, equipped with cost and efficiency advantages [23,68].

## Conclusions

Incorporation of Poland into the European Single Market caused effects of trade creation and diversion for agri-food products, reflected in the increased value of Poland’s trade with the other EU countries, resulting from the elimination of trade barriers within the customs union, as well as substitution of cheaper import from more efficient non-EU countries, import from less efficient, but preferentially treated partner countries. Observed changes led to a high degree of concentration of Polish foreign trade in agri-food products within the European Single Market. Excluding the United Kingdom, which since 2021 has no longer been part of the EU, the share of intraregional trade in the Polish agri-food export increased to over 70%, while in import it reached almost 80%. On the one hand a large share of export onto markets of countries with a stable market economy indicates the capacity for effective export of Polish agri-food products onto foreign markets. However, on the other hand–in view of the limited growth potential of the EU food demand–it provides grounds for the statement that further export expansion will depend to a considerable extent on the capability to expand sales to non-EU markets. In this context it becomes crucial to diversify target markets and search for stable prospective markets characterized by limited non-market risk, located outside the EU.

In the period up to 2021 the USA ranked fourth after the United Kingdom, Ukraine and Russia, as Poland’s non-EU export partner in the agri-food sector, with the beginning of intensive transatlantic cooperation dating back to the 1940s. Although attempts to establish a transatlantic free trade area between the EU and the USA were unsuccessful and protectionist tendencies have strengthened in the US trade policy, in view of the economic potential of both partners and the strength of their impact on the international economic environment, this concept might be implemented in the future. This seems even more probably considering that the aim of the EU trade policy, apart from the reform of the World Trade Organization, providing protection against unfair trade practices and promoting free flow of goods and services on the global scale, is to liberalize trade with the most important trade partners, leading to economic growth in the long-term perspective. In an attempt to determine whether the US market might become a prospective future market for the Polish agri-food sector, facilitating diversification of export onto non-EU markets and identify groups of products possibly serving as export specializations, the aim of this paper was to evaluate the level of comparative advantages of the Polish agri-food sector on the EU and US markets in 2004–2021 using the products mapping technique suggested by Widodo.

Based on the conducted studies it may be stated that in the Polish export of agri-food products onto transatlantic markets food preparations predominated, including primarily preparations of meat, cereals, fruit and vegetables and sugar confectionery, as well as less processed animal origin products such as meat and offal or dairy produce. They are traditional export specialties of Poland, created based on the production potential of the agricultural sector, relative cost and price advantages, as well as quality attributes of agricultural commodities and resulting final products. In trade of these products on both analyzed markets Poland generated high comparative advantages and a positive, systematically improving balance of trade. In turn, Poland had a disadvantageous competitive position in transatlantic trade in products complementary to domestic production, which import was necessary to a considerable extent. On this basis, referring to Ricardo’s classical theory of comparative costs and the neoclassical theory of factor endowment developed by Heckscher, Ohlin and Samuelson we may conclude on rational selection of directions for the realized export specialization.

While in trade with the other EU countries the competitive position of the Polish agri-food sector weakened, it strengthened on the US market. In relation to trade with the EU, which scale is considerably greater, such a trend may cause concern and possibly undermine further development of trade in the region. On the other hand, in view of the high saturation of the EU agri-food market it may be expected that the trade expansion of the Polish agri-food sector will be determined to an increasing degree by the relations with non-EU countries. In this situation due to the need to search for markets alternative to the EU market, this strengthening competitive position on the US market is definitely an advantageous development particularly in the long-term perspective. Although the US market is of minor importance for the agri-food sector in the macro scale due to the relatively limited complementarity of structures of agricultural production in the similar climate zone, it nevertheless is important for at least several branches of the Polish food industry, such as e.g. the meat, fish, confectionery, spirit, tobacco as well as fruit processing industries. When observing the growth rate for export of agri-food products from Poland to the other EU countries and non-EU countries it may be observed that since the 2008–2009 economic and financial crisis trade with non-EU countries has developed more dynamically than the intra-regional trade. On the one hand, such a trend confirms limited potential for further growth of intra-regional trade. On the other hand, it indicates the potential of target markets outside the EU. Poland’s trade with the USA may serve as an example in this respect. Despite problems triggered by the COVID-19 crisis, the growth rate of agri-food export from Poland to the USA in 2021 compared to 2020 was greater than that of export to the other EU countries. Assuming that such a trend may be maintained over a longer time period and especially in the case of reviving the idea of the transatlantic partnership this would significantly increase export of food products from the EU, including e.g. preparations of cereals as well as preparations of vegetables and fruit. Thus, when referring to the more utilitarian value of this study it may be recommended for Poland to encourage policies aiming at maintaining the current export specialization and promoting further development of bilateral trade with the USA to meet institutional requirements of this market and competitive pressure imposed on that market both by US producers and food suppliers from other countries. Specific implications for policy makers can include encouraging tariff liberalization and regulatory cooperation between the EU and the USA enhancing access to the US market and ensuring equal conditions of competition for the EU entities and the US producers. Another important issue is to support the development of long-lasting trade relations and to actively promote Polish agri-food products on the American market in order to create the image of wholesome, attractive and diverse Polish food. Special attention should be paid to organic products and geographical indications.

The USA is one of the potential directions for the diversification of Polish agri-food export outside the EU. Other prospective markets with an increasing economic potential include also markets in Asia, Africa and the Middle East, first of all China, Japan, Vietnam, Australia, New Zealand, Saudi Arabia, Kuwait, Algeria, Morocco, Israel or RSA. The importance of bilateral trade between the EU and many of these countries is shown by the preferential trade agreements either being negotiated or already signed, providing an institutionalized framework for cooperation and eliminating tariff and non-tariff barriers hindering mutual trade. In view of the above, it is advisable to conduct further studies to estimate comparative advantages and to identify these groups of agri-food products, which in the above-mentioned reference markets may become foundations for an advantageous export specialization of Poland. Taking into account the limitation of our study, which is the relatively high degree of data aggregation (the 2-digit HS level), it would be valuable to perform those analyses at the 3-digit level of the UN Standard International Trade Classification (SITC), rev. 3 to offer detailed information on individual products revealing the greatest export potential. Considering present trends in inflation rates and exchange rates, it would also be beneficial to introduce to the analyses constant prices rather than current values in order to offer an objective picture of “real” trade developments and competitiveness.
